# Use of designing for behaviour change framework in identifying and addressing barriers to and enablers of animal source feeding to children ages 8–23 months in Bandarban Hill District in Bangladesh: Implications for a nutrition‐sensitive agriculture programme

**DOI:** 10.1111/mcn.13472

**Published:** 2023-01-06

**Authors:** Md Abul Kalam, Chowdhury A. A. Asif, Ame Stormer, Treena Bishop, Meredith Jackson‐deGraffenried, Aminuzzaman Talukder

**Affiliations:** ^1^ Hubert Department of Global Health, Rollins School of Public Health Emory University Atlanta Georgia USA; ^2^ Helen Keller International Dhaka Bangladesh

**Keywords:** animal source foods, Bangladesh, barrier analysis, behaviour change, behavioural determinants, Chattogram Hill Tracts, child nutrition, designing for behaviour change

## Abstract

Inadequate diet quality is a cause of undernutrition among children 6–23 months of age in Bangladesh, particularly in remote and isolated areas such as Bandarban District. Feeding animal source foods can help to combat stunting and wasting problems among children, but it may not be accessible or acceptable. A barrier analysis using the Designing for Behavior Change Framework was conducted in Bandarban district with participants from 4 ethnic groups, to explore potential barriers and key motivators by examining 12 behavioural determinants of consumption of animal‐source food in complementary feeding for children 8–23 months. Data were collected from 45 mothers of children 8–23 months, who provided animal‐source foods to their children (doers), and from 45 mothers who did not (non‐doers), for a total of 90 interviews. Nine determinants were statistically significantly different between doers and non‐doers as follows: self‐efficacy, positive consequences, negative consequences, social norms, access, reminders, perceived risk, perceived severity and perceived action efficacy. Nearby access to purchase animal‐source foods, rearing poultry or livestock at home and the support of household and community members are enablers to feeding animal‐source food. In contrast, these same factors are barriers for non‐doers. The lack of money to spend on animal‐source foods is also a barrier. An integrated nutrition‐sensitive and gender‐transformative animal‐based food production, and inclusive market programme could increase access to meat and eggs at the household level, increase opportunities to earn income and support gender‐equitable household workloads and decision‐making for optimal child feeding.

## Key messages

- The Designing for Behavior Change Framework is an effective barrier analysis tool to assess and identify food consumption determinants.
- Consumption of animal‐source food for children aged 8–23 months is constrained by the unavailability of ASFs, lack of financial means to purchase, lack of support from household members and perceived negative consequences.
- Consumption of animal‐source food by children aged 8–23 months is enabled by access through household‐level animal rearing, proximity to points of sale, and household and community support.
- An integrated nutrition‐sensitive and gender‐transformative animal‐based food production approach could increase equitable access to and utilization of animal‐source foods.

## 1 INTRODUCTION

Globally, approximately one in five children under 5 years of age (149.2 million) are stunted and 45.4 million children are wasted (Development Initiative, 2021). Asia has the highest rate of stunting (53%) and wasting (70%) globally, and the majority of stunted and wasted children reside in South Asia (UNICEF, WHO & The World Bank, 2021). In Bangladesh, child undernutrition is a public health concern, with 28% of children under 5 years stunted, 25% underweight, and 10% wasted, with children in rural areas suffering from higher rates of undernutrition than in urban areas (NIPORT & ICF, 2019). Inadequate dietary consumption, especially among children 6–23 months, is a primary cause of poor nutrition outcomes for children (Tette et al., 2015). Increasing macro‐ and micronutrient intake, including carbohydrates, protein and fats, as well as vitamins A, B‐12 and riboflavin, calcium, iron and zinc, can improve child growth (Adesogan et al., 2019; Balehegn et al., 2019; Hirvonen et al., 2020; Pasha et al., 2013).

Beyond the age of 6 months, global guidance advises breastmilk alone is not sufficient to provide the required nutrients for children 6–23 months to achieve optimal physical and cognitive development, although continued breastfeeding is recommended in addition to other foods. To ensure and meet nutritional needs, complementary foods should be timely, adequate, safe and responsively fed (Adesogan et al., 2019). Animal‐source foods (ASF) have high concentrations of essential nutrients (Headey et al., 2018; Krebs et al., 2006; Zhang et al., 2016) and consumption of meat (including organ meat), fish, eggs and dairy products (milk, cheese and yoghurt) can support infants and young children to achieve adequate nutrient intake (Robert et al., 2021) and substantially reduce the risk of stunting and wasting (Dewey, 2013).

Children 6–23 months in Bangladesh consume low amounts of animal‐source foods, despite increased meat, milk and egg production in recent years (JPGSPH BRACU & NNS, 2020). Thirty‐nine per cent of breastfeeding children aged 6–23 months were found to have consumed meat, fish or poultry, 36% consumed milk products and another 59% consumed eggs (JPGSPH BRACU & NNS, 2020). Multiple factors, including the mother or caregiver's knowledge of appropriate infant and young child‐feeding practices (Hajeebhoy et al., 2013), availability and access to animal‐source foods (Haileselassie et al., 2020), sociocultural norms about feeding animal‐source foods (Sanghvi et al., 2013), poverty (Adesogan et al., 2019), place of residence (urban or rural) and food security status (Sarma et al., 2017) influence consumption of animal source foods by children 6–23 months.

The remote, hard‐to‐reach Chittagong Hill Tracts (CHTs) region of southwestern Bangladesh is home to 48% of the country's total ethnic minority population with 11 distinct ethnic groups, each with their own language and cultural traditions (Ministry of Health and Family Welfare, 2016). Access to health and nutrition services, education, markets and other services and resources is limited. Most recent data show Bandarban District of the CHT has the highest stunting rate (47.7%) in the country, with underweight and wasting rates of 27.6% and 10.4%, respectively (WFP & IFAD, 2015).

In 2015, a 6‐year multi‐sectoral programme, the United States Agency for International Development (USAID) Resilience and Food Security Activity Sustainable Agriculture and Production Linked to Improved Nutrition Status, Resilience and Gender Equity (SAPLING), was awarded to improve gender‐equitable food security, nutrition and resilience of vulnerable households living in five subdistricts of Bandarban. The programme baseline in 2016 found 34% of children aged 6–23 months had a minimum acceptable diet, with inadequate consumption of animal‐source foods: just 46% of children aged 6–23 months, who were breastfed, consumed flesh foods, 38% consumed eggs and 23% consumed dairy (ICF, 2017). Children's minimum acceptable diet was higher in households above the poverty line and where the head of household completed primary education or higher. The baseline also found varying beliefs regarding feeding young children protein‐rich animal‐source foods, such as fish or other meat. Little additional information is available on children's nutritional trends in this remote, multi‐ethnic area, including the consumption of animal‐source foods. To inform programme implementation for improving child nutrition, the primary objective of this study was to explore the multi‐dimensional behavioural determinants of animal‐source foods in complementary feeding for children aged 8–23 months in the programme area.

## 2 MATERIALS AND METHODS

### 2.1 Conceptual framework

The study used the Designing for Behavior Change (DBC) framework (The TOPS Program, 2016) to identify and address the barriers to ASF feeding practices. The DBC is a comprehensive tool that helps to achieve better and sustainable behaviour change results by guiding programme designers through five key components as follows: (i) behaviour, (ii) priority group or influencing group, (iii) determinants, (iv) bridges to activities and (v) activities. In the DBC approach, based on the health belief model and theory of planned behaviour (Kalam, Davis, Shano, et al., 2021), barrier analysis (BA) is used to identify determinants, influencing groups and inform activities for behaviour change. The BA is a rapid assessment tool using doer/non‐doer methodology that identifies the barriers and enablers, and the most important behavioural determinants associated with a particular behaviour (Kalam, Davis, Islam, et al., 2021). Comparative data are collected from doers (individuals who perform the behaviour) and non‐doers (individuals who do not perform the behaviour) using both open‐ended and close‐ended questions (Davis, 2010; Gibbs & Collett, 2016; The TOPS Program, 2016). The BA has a predetermined list of 12 evidence‐based determinants for health and nutrition behaviours (see Supporting Information: File 1).

### 2.2 Questionnaire development and pretest

The DBC questionnaire and prescribed analysis are designed to identify the most likely behavioural determinants of the specified study behaviour. In this study, the behaviour was defined as ‘Mothers of children ages 8–23 months feed their children animal source foods every day’. The decision to include mothers of children 8–23 months and not 6–23 months was made, because mothers will have had at least 2 months of experience practising complementary feeding (if following guidance to start at 6 months) from which to draw on for responses, whereas mothers of children 6 months of age would just be introducing foods other than breastmilk to the children's diet. The questionnaire for this BA contained two sections (see Supporting Information: File 1). Section A is a set of behaviour screening questions to determine if the eligible participants are doers or non‐doers, based on their practices with regard to complementary feeding behaviours. Section B contains behavioural questions designed to provide information on the preselected determinants being studied. Questions were developed by the study team in English following DBC BA determinants. The questionnaire was reviewed by the study team members and a DBC BA expert, translated into Bangla, and then backtranslated into English to ensure accuracy. The questionnaire was pretested in different paras (villages) of Bandarban Sadar subdistrict to ensure the appropriateness of the questions and suitability of the language, and to provide data collectors with practical field experience in data collection. Based on the pretest, slight modifications were made to ensure linguistic clarity. The pretest also helped the data collection team to have a better understanding of the questionnaire.

### 2.3 Sampling strategy, inclusion criteria, recruitment and data collection

The BA approach requires 90 individual interviews with 45 doers and 45 non‐doers to find statistically significant results (Kittle, 2017). Thus, 90 respondents were recruited from all five subdistricts (Bandarban Sadar, Rowangchari, Lama, Ruma and Thanchi) of Bandarban District, who were participating in the SAPLING project. Although there are 10 ethnic groups residing in the programme area, the BA sampling strategy prioritized geographic diversity and did not stratify by ethnic group. In each of the five subdistricts, respondent villages with a minimum population of 60 households were identified, resulting in a list of 47 eligible villages. From this list, 3 villages per subdistrict were selected randomly for a total of 15 villages, and 3 doers and 3 non‐doers were interviewed for a total of 9 doers and 9 non‐doers in each subdistrict.

### 2.4 Ethics approval and data collection

The study was conducted according to the guidelines laid down in the Declaration of Helsinki and its later amendments. Under the Department of Health and Human Services regulations found at 45 CFR 46.101(b)(2), Chesapeake Internal Review Board (IRB) granted an exemption to IRB oversight for this research (Pro00019477) through the e‐system Center for Institutional Review Board Intelligence. Data collectors were informed of the study objectives before taking part in the interviews. A written script was read to each participant before starting the interview. Written consent was recorded for each participant to reflect their willingness to participate in the study. Data collection took place on 8–11 December 2017, using paper‐based questionnaires. Frontline staff from SAPLING were recruited to collect the data under the supervision of project supervisors. These staffs were recruited from the communities where data were collected, because they were trusted by the community and know the local dialect where they work.

### 2.5 Data management and analysis

Data analysis was completed in three steps. First, data were deductively coded according to each preselected determinant. Responses to open‐ended questions under each determinant were inductively coded into themes or categories by a three‐person team. An analysis workshop was conducted to code open‐ended data and inter‐rater reliability was undertaken through discussion and agreement in the workshop by the three coders. Responses to closed‐ended questions were automatically coded to the response codes. The second step involved tabulation: once coding was completed, the team tabulated the results (number and percentage of doers and non‐doers) to each question by recording the category frequencies in a preformulated Tabulation Sheet (in MS Excel) that uses estimated relative risk (ERR), considering the prevalence of the behaviour in the population to formulate statements of association. The sheet also keeps results and prevalence separate for doers and non‐doers for a comparative analysis. The tabulation sheet is preformulated to determine the statistical significance (*p* ≥ 0.05) between doers and non‐doer responses. The final step involved interpreting results: for example, looking at the ERR of the significant responses between doers and non‐doers, examining whether barriers mentioned by doers may not have kept them from doing the behaviour, looking at enablers that were mentioned by non‐doers more than doers.

Additional analysis has also been conducted to explain the implications of this study on future nutrition‐sensitive interventions. We analysed monthly routine participant survey databases from 2017 to 2020. A list of enrolled participants was developed and maintained for each SAPLING activity, including the Integrated Enhanced Homestead Food Production (IEHFP) and Maternal Child Health and Nutrition (MCHN) groups, and considered as the sampling frame. Using a stratified cluster sampling approach, each sampling frame was stratified by the union and within each union, 72 households (HHs) were randomly selected for the IEHFP survey and 24 HHs selected for the MCHN survey. This totaled 1872 HHs for IEHFP and 624 HHs for MCHN have been selected and surveyed in each fiscal year.

## 3 RESULTS

Among the women interviewed, 8 of the 12 determinants emerged as statistically significant (*p* < 0.05) between doers and non‐doers for feeding ASF to children 8–23 months of age: perceived positive consequences, perceived negative consequences, perceived self‐efficacy, access, cues for action, susceptibility of risk, perceived severity, and action efficacy. In the following descriptions, significant results are presented, whereas other results can be found in Supporting Information: File 2. Table 1 displays all statistically significant results by the determinant.

**Table 1:** Significant results, by determinant

Determinants	Doers *n* (%)	Non‐doers *n* (%)	Diff.	Odds ratio	95% CI	ERR
Self‐efficacy: What makes it easier to feed animal source foods?						
Livestock and poultry rearing at home	22 (49)	11 (24)	24%	2.96*	1.21–7.25	2.60
When animal‐source foods are available at home	25 (56)	12 (29)	29%	3.44**	1.42–8.32	2.98
When household members and husband support	18 (40)	9 (20)	20%	2.67*	1.04–6.85	2.36
When money is available	8 (18)	16 (36)	18%	0.39*	0.15–1.04	0.42
Perceived self‐efficacy: Makes it difficult to feed animal source foods?						
Difficult when money is not available	2 (4)	25 (56)	−51%	0.04**	0.01–0.17	0.05
Difficult when child is sick	23 (51)	8 (18)	33%	4.84**	1.85–12.65	3.91
When market is far from home	1 (2)	10 (22)	−20%	0.08**	0.01–0.65	0.09
When the foods are not available at home	1 (2)	11 (24)	−22%	0.07**	0.01–0.57	0.08
Perceived positive consequences: What are the advantages of feeding animal source foods?						
Boosts physical development	43 (96)	21 (49)	49%	24.57*	5.30–113.93	20.20
Keeps healthy	35 (78)	22 (29)	29%	3.66**	1.47–9.13	3.26
Fewer illnesses	21 (47)	12 (27)	20%	2.41*	1.00–5.82	2.18
Children can walk and move early	16 (36)	8 (18)	18%	2.55*	0.96–6.79	2.27
Mothers can concentrate on their work	8 (18)	2 (4)	13%	4.65*	0.93–23.27	3.53
Perceived negative consequences: What are the disadvantages of feeding animal source foods?						
No negative consequences	37 (82)	24 (53)	29%	4.05**	1.55–10.60	3.60
Having diarrhoea and stomach upset	5 (11)	15 (33)	−22%	0.25**	0.08–0.76	0.28
Having fever and cough	8 (18)	2 (4)	14%	4.65*	0.93–23.27	3.53
Social norms: Who are the people that approve you to feed animal‐source foods?						
Father‐in‐law	3 (7)	11 (24)	−18%	0.22*	0.06–0.86	0.24
Other family members	44 (98)	25 (56)	42%	35.20**	4.45–278.26	29.61
Perceived access: How difficult it is to get ASFs you need to feed your child?						
Very difficult	4 (9)	28 (62)	−53%	0.06**	0.02–0.19	0.07
Not difficult at all	24 (53)	4 (9)	44%	11.71**	3.59–38.20	7.43
Cues for action/reminder: When you prepare meals for your child, how difficult is it/would it be to remember to include foods from these food items?						
Very difficult	1 (2)	7 (16)	−13%	0.12**	0.01– 1.05	0.00
Somewhat difficult	13 (29)	22 (49)	−20%	0.42*	0.18–1.01	0.46
Not difficult at all	31 (69)	15 (33)	36%	4.43**	1.83–10.73	3.79
Perceived risk: How likely is it that your child will become sick/malnourished in the coming year?						
Very likely	1 (2)	9 (20)	−18%	0.09**	0.01– 0.75	0.10
Not likely at all	22 (49)	7 (16)	33%	5.19**	1.92–14.06	4.11
Perceived severity: How serious would it be if your child became sick/malnourished?						
Very serious	6 (13)	17 (38)	−24%	0.25**	0.09–0.72	0.28
Not serious at all	12 (27)	2 (4)	22%	7.82**	1.64–37.36	5.09
Action efficacy: How likely is it that your child would become sick/malnourished if you feed him/her animal‐source food each day?						
Very likely	1 (2)	6 (16)	−14%	0.12**	0.01–1.05	0.03
Somewhat likely	9 (20)	21 (47)	−27%	0.29**	0.11–0.73	0.32
Not likely at all	30 (67)	6 (13)	53%	13.00**	4.51–37.51	8.71

Abbreviations: CI, confidence interval; ERR, estimated relative risk.

* *p* > 0.05

** *p*>0.01.

### 3.1 Perceived self‐efficacy: Easy or difficult to feed animal‐source food each day

Self‐efficacy included questions on what makes it easier and difficult (doer) or would make it easier and difficult (non‐doer) to feed children animal‐source food each day. Responses to these self‐efficacy questions revealed access, availability and family assistance to be enablers for doers and barriers for non‐doers to having the ability to feed animal‐source foods to children. Specifically, doers were 2.6 times more likely to say rearing livestock and poultry in the household makes ASF easy, because it provides eggs, meat and milk (*p* = 0.014). They were further three times more likely to say feeding is easy, because animal‐source foods are available at home (*p* = 0.005). Doers were also 2.4 times more likely to say support from family members (by purchasing, collecting, cooking and feeding these foods to the children) (*p* = 0.032) made it easy. Conversely, non‐doers were 2.4 times more likely to say having money in hand would make ASF easy (*p* = 0.047).

The above‐mentioned enablers are found to be barriers for non‐doer mothers, because they do not have those facilities. Non‐doers voiced significant challenges: they were 21.9, 11.2 and 12.6 times more likely to say not having money to purchase ASF (*p* = 0.000); a market is not available nearby (*p* = 0.004); and unavailability at home (*p* = 0.002) made it difficult to provide ASF to children. For doers, however, feeding animal‐source food becomes difficult when children are sick (doers are 3.9 times more likely to say this; *p* = 0.001).

### 3.2 Access: How easy it is to get animal‐source food to feed to children daily

Perceiving ease or difficulty to obtain or access animal‐source foods influences the behaviour. Non‐doers were 13.5 times more likely to say it is difficult to obtain animal‐source foods to feed their children (*p* = 0.000), whereas doers were 7.4 times more likely to say it is not difficult (*p* = 0.000). The responses to the perceived self‐efficacy questions presented above elaborate that non‐doers said it would be easier to feed animal‐source food if they reared livestock at home and if they had a market or vendor near their home. Likewise, doers said these two things made it easier for them to feed animal‐source food to their children.

### 3.3 Positive and negative consequences: Advantages and disadvantages of feeding animal‐source foods regularly

Doers were more likely to say children benefit from animal‐source food in terms of physical, cognitive, and motor skills, and immune system development. Although non‐doers were less likely to be aware of the advantages than doers, at least half of non‐doers demonstrated knowledge of the benefits of feeding animal‐source food daily, despite not practising this behaviour. Specifically, doers were 20.2 times more likely to say animal‐source food boosts physical development (*p* = 0.000), 2.3 times more likely to say it boosts motor skill (walking and moving) development (*p* = 0.047), 3.3 times more likely to say it keeps children healthy (*p* = 0.004), and 2.2 times more likely to say children get fewer sicknesses/illnesses when they consume animal‐source foods (*p* = 0.040).

Regarding perceived negative consequences of giving ASF, doers were 3.6 times more likely to say there were no disadvantages to feeding it to children (*p* = 0.003). Non‐doers were 3.6 times more likely than doers to say children get diarrhoea and stomach upset due to giving ASF (*p* = 0.010), whereas doers (18%) were more likely to say children develop fever and cough as a result of giving ASF (*p* = 0.045).

### 3.4 Cues for action: How difficult it is to remember to feed animal‐source food daily

Cues for action appeared to be a major barrier for the non‐doers. They were 3.8 times more likely to say it would be difficult to remember to feed ASF daily (*p* < 0.001), whereas doers are 3.8 times more likely to say it is not difficult to remember (*p* < 0.001).

### 3.5 Susceptibility of risk: Likelihood that children will become malnourished

Perceived risk of becoming malnourished and the level of concern about getting sick also appeared to be a predictive determinant of feeding with animal‐source foods. Non‐doers were 2.5 times more likely to believe their children will be malnourished in the coming year (*p* = 0.023) Conversely,doers were 4.1 times more likely to say it is not likely at all their children will be malnourished in the coming year (*p* = 0.001).

### 3.6 Perceived severity: Extent of severity if children become malnourished

The perceived severity of malnourishment was also predictive of feeding animal‐source foods to children. Non‐doers were 2.1 times more likely to believe the occurrence of being sick or malnourished in the coming year will be very or somewhat serious (*p* = 0.007), whereas doers were 5.1 times more likely to say the occurrence would not be serious at all (*p* = 0.004).

### 3.7 Action efficacy: Likelihood that feeding animal‐source goods daily to children will cause children's sickness or malnourishment

Action efficacy differs from the susceptibility to risk in that it assesses the perception that the specific behaviour could cause malnourishment or sickness rather than assessing the perceived risk of becoming malnourished or sick from any cause. Doers were 8.7 times more likely to say feeding ASFs daily would not cause children to be sick or malnourished in the coming year (*p* = 0.000). Conversely, non‐doers were 5.1 times more likely to say it was somewhat or very likely that feeding ASFs would cause children to become sick or malnourished (*p* = 0.000).

### 3.8 Perceived social norms: Who approves or disapproves of feeding ASF daily

There were no significant differences between doers and non‐doers for perceived social norms being an enabler or barrier, as they both said no one disapproves of feeding ASFs. Regarding the approval, non‐doers were 4.1 times more likely to mention their father‐in‐law would approve of the behaviour (*p* = 0.019), whereas doers were 29.6 times more likely to say that their intermediate family members (i.e., extended family network) approve of this behaviour (*p* = 0.002).

## 4 DISCUSSION

This DBC BA study identified eight significant barriers and enablers for mothers of children 8–23 months in the Bandarban District of the CHT region of Bangladesh to feed ASFs daily to their children. Due to household poverty, geographical remoteness and isolation, access to animal‐source foods emerged as the most critical determinant in the study population. Three factors were found to directly impact access: (1) rearing poultry and livestock at home, (2) availability of a nearby place to purchase animal‐source foods, and (3) not having the financial resources to purchase animal‐source foods. The study highlighted that limited availability to purchase and not rearing animals at home was perceived as a barrier by non‐doers, which aligns with findings from other studies in other rural areas of Bangladesh (Rasheed et al., 2011; Thorne‐Lyman et al., 2017). In addition, lower purchasing power (i.e., not having money on hand) was also seen as a barrier to accessing animal‐source foods, confirming findings from previous studies in Bangladesh (Affleck & Pelto, 2012; Mukta et al., 2015; Rasheed et al., 2011) and other countries (Burns et al., 2016; Colecraft et al., 2006; Wong et al., 2020).

Although not having a place to purchase animal‐source foods nearby, not having money to purchase them, and not rearing them at home are barriers for the non‐doers, these same factors were the enablers for doers who said having market access and rearing at home made it easy to feed animal‐source foods to their children 8–23 months. Mothers who do not feed animal‐source foods to their children said having this access would enable them to adopt this practice. Ownership of poultry and livestock has been previously shown to be associated with young child consumption of animal‐source foods in Bangladesh (Choudhury & Headey, 2018; Talukder et al., 2010), Ethiopia (Kim et al., 2019a), Tanzania (Bundala et al., 2020; Kim et al., 2019) and the Sahel (Dominguez‐Salas et al., 2019). The global analysis of the EAT–Lancet reference diet showed that consuming the recommended diet, including animal‐source foods, is not affordable for most people in low‐ and middle‐income countries (Hirvonen et al., 2020). Other studies conducted in Bangladesh (Affleck & Pelto, 2012; Mukta et al., 2015; Rasheed et al., 2011; Thorne‐Lyman et al., 2017), Sub‐Saharan Africa (Hetherington et al., 2017), and Ethiopia (Haileselassie et al., 2020) have similarly found lack of affordability to be a barrier. Conversely, even those with access to poultry and livestock may have issues of availability as poultry, and livestock may be viewed as a source of income or asset and not a food source; households that rear poultry and other livestock may choose to sell their animals rather than consume them. In Timor‐Leste, results indicated that individuals chose to sell their animal products to earn income, because the products are not considered food, but rather an asset (they found a perception that ‘eating meat’ means ‘eating money’) (Wong et al., 2020).

In addition to access, support from household members, including husbands and mothers‐in‐law, and other community members emerged as a potential key enabler to feeding animal‐source foods to children 8–23 months (it should be noted that not having support was not a significant barrier). Having a household member who supports appropriate child feeding could mitigate the potential negative effects on child diet that mothers' heavy workloads may have. In Bangladesh and other low‐ and middle‐income countries, women suffer from severe time constraints and bear the burden of responsibility for household chores and primary care of children and other household members (Sinharoy et al., 2018; Sraboni & Quisumbing, 2018). In the CHTs, engagement in traditional cultivation, often in locations away from the house, and other income activities, which require labour and time, exacerbates already heavy workloads for women (Kabir et al., 2019; Uddin, 2014). Mothers’ heavy workloads and lack of support from husbands and other family members have been found to negatively impact appropriate complementary feeding in Bangladesh (Affleck & Pelto, 2012; Rasheed et al., 2011).

Other factors from the sociocultural context also influence child feeding. Some mothers in this BA said elders advise against feeding animal‐source foods to children, which may be due to beliefs about negative consequences. Previous studies in Bangladesh found that mothers‐in‐law and elderly community members, neighbours and community health workers influence decisions regarding the child and maternal nutrition behaviours (Avula et al., 2013; Hoddinott et al., 2017; Kim et al., 2018).

The sociocultural context influences child feeding practices (Boak et al., 2016; Monterrosa et al., 2012) and related norms, which inhibit feeding animal‐source foods to children due to perceived negative consequences can also be a barrier, whereas perceptions of positive consequences can be an enabler. For mothers who do not feed animal‐source foods to their children under 2 years, diarrhoea, upset stomach, cough and fever were perceived as negative consequences. The idea that animal‐source foods can cause digestive issues is found across the world. The Alive and Thrive programme in Bangladesh and Ethiopia observed similar perceptions in which mothers thought children cannot digest meat and other animal products, which can cause sickness, including vomiting and stomach upset (Alive & Thrive, 2010; Mukta et al., 2015). Similarly, Mexican female caregivers reported beef is not given to young children, because they are unable to digest it (Pachón et al., 2007). In this BA, mothers who fed animal‐source foods to their children did not think it likely that the foods would cause their child to become sick or malnourished and reported it boosts children's physical growth, immune function, child health and motor skills. In contrast, mothers who do not feed animal‐source foods not only were more likely to think these foods could cause their child to become sick or malnourished but also had a perception that if a child becomes sick, it is divine will (see Supporting Information: File 2) and out of their hands. If a child does become sick, all mothers find it difficult to feed animal‐source foods to their children due to illness or lack of appetite.

## 5 IMPLICATIONS FOR NUTRITION‐SENSITIVE PROGRAMMES

Based on the findings of the BA, the SAPLING programme designed behaviour change activities following the DBC framework. The key strategies included increasing access to ASF, increasing the perception that ASF can ensure a child's physical and cognitive benefits, ensuring support of household members, increasing the ability to remember to feed ASF and empowering women to engage in future‐oriented behaviour. SAPLING provided training to pregnant and lactating women and women from all poor and extremely poor HHs on IEHFP, which includes poultry rearing and the importance and benefits of ASF. This activity provided access to eggs and meat at the HH level, while also increasing income through selling surplus. Additionally, poultry rearing is an Income Generating Activity supported by SAPLING that contributed to the availability of ASFs such as eggs and meat in the local markets. Separate sessions for senior women and men were designed to increase knowledge and awareness of child nutrition and also include messages on ASF to address the perceived negative consequences and perceived risks. A wider communication strategy included activities to support the adoption of these promoted practices, including billboards, community theatre, and videos. Cooking demonstrations were held with participants to show them recipes to improve dietary diversity for children, including ASF. SAPLING also promoted women's empowerment through a gender‐transformative approach to more equitably divide workloads, including childcare and child feeding, by strengthening relationships, as well as ensuring all members of the household have knowledge and skills for appropriate complementary feeding.

The combination of poultry‐rearing activities, Essential Nutrition Action sessions and gender sensitization increased access to ASF and children's consumption of animal‐source foods. Survey results demonstrated that households that were trained in poultry production increased the average number of birds from 7.1 to 14.1 from 2017 to 2020 (Table 2).

**Table 2:** Results on poultry production and ASF consumption by children aged 8–24 month^a^

	Year‐17	Year‐18	Year‐19	Year‐20
Average number of poultry birds reared by households	7.1	7.5	8.9	14.1
ASF consumption by type by year (%)				
Organ meat	3	13	18	21
Other meat	8	18	22	23
Eggs	47	59	61	63
Large fish	18	20	27	24
Small fish	10	29	27	28
Dairy	14	18	18	27

Abbreviation: SAPLING, Sustainable Agriculture and Production Linked to Improved Nutrition Status, Resilience and Gender Equity.

^a^ This result extracted from the routine participant survey from 2017 to 2020. The survey was conducted with a subsample of 624 of the SAPLING programme.

While looking at the children's ASF consumption data, overall, different types of ASFs consumption increased gradually over the programme duration (Table 2). While looking at the results by type of ASFs, it was revealed that organ meat and small fish consumption increased by 18 percentage points, followed by eggs and other meats (16 and 15 percentage points, respectively) from 2017 to 2020. This strategy also aligned with other studies that found ownership of poultry and livestock is associated with young child consumption of animal‐source foods in Bangladesh (Choudhury & Headey, 2018; Talukder et al., 2010), Ethiopia (Kim et al., 2019a), Tanzania (Bundala et al., 2020; Kim et al., 2019) and the Sahel (Dominguez‐Salas et al., 2019).

Two study limitations should be noted. First, due to the small, targeted sample of this BA, the results cannot be generalized to the population level but are representative of the largest villages in five districts of Bandarban served by SAPLING and its USAID‐funded follow‐on project, the Bandarban Agriculture and Nutrition Initiative (BANI). The results were used to inform project interventions. SAPLING and BANI developed an evidenced‐based social and behaviour change communication strategy to address the barriers and leverage the enablers in promoting ASF practices. This approach could also be replicated in other nutrition‐sensitive interventions to determine how to best promote animal source foods and their benefits.

Second, mothers of children 8–23 months were selected so that other barriers and enablers perceived by other influential groups, such as mothers‐in‐law, husbands and community health service workers, may have been missed.

## 6 CONCLUSION

Animal‐source foods are key sources of essential nutrients for children 8–23 months, yet consumption of these foods among this age group is low in Bangladesh. This study was the first of its kind to highlight critical barriers and enablers for mothers from diverse ethnic communities in the Bandarban District of the CHT region of Bangladesh to feed animal‐source foods to their children. The results add to global evidence that access to promoted foods—both physical access and financial access, household and community support for mothers, and sociocultural norms are key behavioural determinants to adopting optimal child‐feeding practices. Integrated programmes, which combine nutrition‐sensitive animal husbandry, diversified livelihoods, inclusive markets and a transformative gender approach, can support families to improve child feeding while balancing daily livelihood needs and responsibilities. As evident in survey findings, rearing poultry and other livestock at home combined with nutrition education and skills is an integrated livelihood and nutrition strategy that could increase consumption of animal‐source foods for children 8–23 months by eliminating the cost of purchasing the foods, facilitating access to animal‐source foods, facilitating access to income and improving understanding and awareness of the importance of household and child consumption. Household and community members are potential agents of change who can play a significant role in adopting appropriate feeding behaviours for young children. Finally, strengthening knowledge and skills for appropriate complementary feeding for all household members and community members, such as health service providers and vendors, as well as increasing access through income generation and availability in nearby points of sale, can form a holistic approach, which enables feeding animal‐source foods to children 8–23 months.

## AUTHOR CONTRIBUTIONS

Md Abul Kalam, Chowdhury A. A. Asif, and Meredith Jackson‐deGraffenried designed the study. Md Abul Kalam led the investigation. Md Abul Kalam and Meredith Jackson‐deGraffenried designed the analysis plan. Md Abul Kalam conducted the analysis. Md Abul Kalam and Chowdhury A. A. Asif drafted the manuscript. Ame Stormer, Meredith Jackson‐deGraffenried, Treena Bishop and Aminuzzaman Talukder reviewed and edited the manuscript. Meredith Jackson‐deGraffenried and Aminuzzaman Talukder supervised the study. All authors read and approved the final manuscript.

## CONFLICT OF INTEREST

The authors declare no conflict of interest.

## ETHICS STATEMENT

The current study was conducted according to the guidelines laid down in the Declaration of Helsinki and its later amendments. Under the Department of Health and Human Services regulations found at 45 CFR 46.101(b)(2), Chesapeake Internal Review Board (IRB) granted an exemption to IRB oversight for this research (Pro00019477) through the e‐system Center for Institutional Review Board Intelligence (CIRBI). Written consent was recorded for each participant to reflect their willingness to participate in the study. No identifiable information was collected from the respondents.

## Supporting information

Supporting information.

Supporting information.

## Data Availability

The data that support the findings of this study are available in Supporting Information: File 2.
