# Supplementary material for: Use of designing for behaviour change framework in identifying and addressing barriers to and enablers of animal source feeding to children ages 8–23 months in Bandarban Hill District in Bangladesh: Implications for a nutrition‐sensitive agriculture programme
Source: Matern Child Nutr. 2023 Jan 6;19(2):e13472. doi: 10.1111/mcn.13472 (PMC10019048; doi:10.1111/mcn.13472)
Supplement: Supplementary file 1 — Supporting information. [file MCN-19-e13472-s001.docx]

**Details of the study determinants**

| **Key terms** | **Definition** |
| --- | --- |
| Study behavior | Mothers of children ages 8 – 23 months feed their children animal source food each day during meals. |
| Target group | Mother of children 8-23 months |
| **Behavioral determinants** | |
| Perceived self-efficacy | An individual’s belief that he/she can do a particular behavior given his/her current knowledge, resources and skills. |
| Perceived social norms | The perception that people important to an individual think that he/she should do the behavior (injunctive norms), and plan to do the behaviors (descriptive norms). |
| Perceived positive consequences | What positive things a person thinks will happen as a result of performing a behavior. |
| Perceived negative consequences | What negative things a person thinks will happen as a result of performing a behavior. |
| Access | The degree of availability of the needed facilities, services or materials required to adopt the behavior. |
| Cues for action | The presence of reminders that help a person remember to do a particular behavior. |
| Perceived susceptibility/risk | A person’s perception of how vulnerable or at risk they feel vis-à-vis the problem or disease. |
| Perceived severity | Belief that the problem or disease (which the behavior can prevent) is serious. |
| Perceived action efficacy | The belief that by practicing the behavior one will avoid the problem or disease; that the behavior is effective in preventing the problem or disease. |
| Perceived divine will | A person’s belief that it is God’s / Allah’s or the gods’ will (depending on their faith) for him/her to have the problem and/or to overcome it. |
| Policy | Laws and regulations (local, regional, or national) that affect adoption of the behavior and access to products and services. |
| Culture | The set of history, customs, lifestyles, values, and practices within a self-defined group. |

**Barrier Analysis Questionnaire:**

| **Scripted Introduction:**  Hi, my name is ; and I am working in SAPLING program that is being implemented by Helen Keller International with support from other local NGOs. I am part of a study team looking into child feeding practices including animal source foods. Before I continue, I would like to know the age of your youngest child. (*If the child is in the desired age range, read out the following script and get consent before starting the interview [see question 1]. If the child is not in the desired age range, thank the mother and look for another respondent.)*  The study includes a discussion of feeding practices and will take about 25-30 minutes. I would like to hear your views on this topic.  Your participation in this study is completely voluntary and you are free to decide whether you participate in the study or not. If you decide to participate, you can withdraw your participation at any point of the interview. You have the full freedom to not answer any specific question if you do not want to answer.    Please know that any information you provide will be kept confidential and used only for research purpose and will not be used any way that can identify you. No harm will be occurred as a result of participation in the study except spending some time voluntarily. Your all responses, notes and records will be kept in a secured location by using a sealed locker. Only the research team members will have the access on those for analysis purpose.  The results of this study will be used exclusively for the purpose of the study; specifically, to inform program to design and delivery better services, report preparation and journal publication without jeopardizing your identification. As we are not asking you to provide any identification information other than your name, there is no chance to reveal your identity not identify you as a result of report or journal publication. However, for the written consent purpose, we need to know your name and for the data collection tracking purpose, we need to know your village name. We will remove the page containing your name and your village information before analyzing your responses. So, we are ensuring your privacy and confidentiality.  Would you like to participate in the study? [If not, thank them for their time.] [If agree to participate, please explain:]. By signing below and returning this form, you are consenting to participate in this study.  Respondent name: …………………….  Signature: ………………….  Date: …………………………  Interviewer name: …………………………  Signature: ……………….  Date: ……………………… |
| --- |

Questionnaire No.: Date: ____/ / Para: Union: Ethnicity: ____

**Section A - Doer/Non-doer Screening Questions**

1. How old is your youngest child? _____________(Write the age in months here)
   1. 8 months - 24 months

b. 7 months or younger>>>> *End interview and look for another mother*

c. Older than 24 months>>> End interview and look for another mother

d. Don’t Know / Won’t say>>> *End interview and look for another mother*

1. I would like to you think about all the food items you fed your baby in the last 2 days. What type of foods did you feed your baby something other than breast milk? *(This question is just to help the mother to remember what the baby ate.)*
   1. (foods name)
   2. Do not know / no response>>*End interview and look for another respondent}*
2. Please tell me all the different foods you remember feeding to your baby in the last two days. (*If the mother mentions a dish that has several ingredients, ask her to list them all*. *Check all the boxes of foods the mother mentions*.)
3. Do not know / no response>>>*End interview and look for another respondent*
4. Dairy products: [milk, yogurt or milk made any product]
5. Flesh foods: [frog, small fish, large fish, crab, dry fish, Nappi, chicken, liver, pork, beef, snail, squirrel] [list would vary according to the community]
6. Eggs
7. Others [anything related to animal source, according to the community]

# DOER /NON-DOER CLASSIFICATION TABLE

| **DOER**  (all of the following) | **Non-Doer**  (any ONE of the following) | **Do Not Interview**  (any ONE of the following) |
| --- | --- | --- |
| Question 1 = A |  | Question 1 = B or C or D |
| Question 3 = **two** or more boxes checked from C through d | Question 3 = one **or none** boxes checked from C through E | Question 3 = A |

**Select a group based on the table:** **☐ Doer.** **☐ Non-doer.**

**Section B – Research Questions**

*(Perceived Self-efficacy)*

***1a. Doers:*** What makes it **easy** for you to feed your baby from these (animal source) food items each day? Why?

**1b. *Non-doers:*** What would make it ***easy*** for you to feed your baby from these (animal source) each day? Why?

*(Write all responses below. Probe with “What else?”)*

**2a. *Doers:*** What makes it ***difficult*** for you to feed your baby from these food items (animal source) each day? Why?

**2b. *Non-doers:*** What would make it ***difficult*** for you to feed your baby from these food items (animal source) each day? Why?

*(Write all responses below. Probe with “What else?”)*

*(Perceived Positive Consequences)*

**3a. *Doers:*** What are the **advantages** of feeding your baby from these (animal source) food items each day? Why?

**3b. *Non-doers:*** What would be the **advantages** of feeding your baby from these (animal source) food items each day? Why?

***(****Write all responses below. Probe with “What else?”)*

*(Perceived Negative Consequences)*

***4a. Doers:*** What are the **disadvantages** of feeding your baby from these (animal source) food items each day? Why?

**4b. *Non-doers:*** What would be the **disadvantages** of feeding your baby from these (animal source) food items each day? Why?

*(Write all responses below. Probe with “What else?”)*

*(Perceived Social Norms)*

**5a. *Doers:*** Who are the people that ***approve*** of you feeding your baby from these food items (animal source) each day?

**5b. *Non-doers:*** Who are the people that ***would approve*** of you feeding your baby from these food items (animal source) each day? Why would they approve?

*(Write all responses below. Probe with “Who else?”)*

**6a. *Doers:*** Who are the people that ***disapprove*** of you feeding your baby from these food items (animal source) each day? Why do they disapprove?

**6b. *Non-doers:*** Who are the people that **would disapprove** of you feeding your baby from these food items (animal source) each day? Why would they disapprove?

*(Write all responses below. Probe with “Who else?”)*

*(Perceived Access)*

**7a. *Doers:*** How difficult is it to get all of these items? Would you say it is Very difficult, somewhat difficult or not difficult at all?

- - - a. Very difficult
    - b. Somewhat difficult
    - c. Not difficult at all
    - d. Do not know

**7b. *Non-doers:*** How difficult would it be to get all of these items? Would you say it is Very difficult, somewhat difficult or not difficult at all?

- - - a. Very difficult
    - b. Somewhat difficult
    - c. Not difficult at all
    - d. Do not know

*(Perceived Cues for Action / Reminders)*

**8a. *Doers:*** When you prepare meals for your baby, how difficult is it to **remember** to include foods from these food items?

- - - a. Very difficult
    - b. Somewhat difficult
    - c. Not difficult at all
    - d. Do not know

**8b. *Non-doers:*** When you prepare meals for your baby, how difficult do you think it **would be to remember** to include foods from these food items? *Very difficult, somewhat difficult, or not difficult at all?*

- - - a. Very difficult
    - b. Somewhat difficult
    - c. Not difficult at all
    - d. Don’t Know

*(Perceived Susceptibility / Perceived Risk)*

***9. Doers and Non-doers*:** How likely is it that your child will become sick/ malnourished in the coming year? Very likely, somewhat likely, or not likely at all?

- - a. Very likely
  - b. Somewhat likely
  - c. Not likely at all
  - d. Do not know

*(Perceived Severity)*

***10. Doers and Non-doers*:** How serious would it be if your baby became sick/ malnourished? A very serious problem, somewhat serious problem, or not serious at all?

- - a. Very serious problem
  - b. Somewhat serious problem
    - c. Not serious at all
    - d. Do not know

*(Action Efficacy)*

***11. Doers and Non-doers*:** How likely is it that your baby would become sick/ malnourished if you feed him/her foods from these food items (animal source) each day? Very likely, somewhat likely, not very likely?

- - a. Very likely
  - b. Somewhat likely
    - c. Not likely at all
    - d. Do not know

*(Perception of Divine Will)*

**12. *Doers and Non-doers:*** Do you think that God causes children to become malnourished?

- - a. Yes
  - b. Maybe
    - c. No
    - d. Do not know

*(Culture)*

***13. Doers* and Non-doers:** Are there any cultural rules or taboos that you know of against feeding your baby from these food items each day?

- - a. Yes
  - b. Maybe
    - c. No
    - d. Do not know

*(Policy)*

***14. Doers* and Non-doers:** Are there any policy that you know that support feeding your baby from these food items each day?

- - a. Yes
  - b. Maybe
    - c. No
    - d. Do not know

***THANK THE RESPONDENT FOR HIS OR HER TIME!***
